# Inhibition of NO-synthase and degranulation of rat omental mast cells *in vitro*

**DOI:** 10.1155/S0962935196000361

**Published:** 1996-09

**Authors:** A. M. Northover, B. J. Northover

**Affiliations:** 1 Department of Pharmaceutical Sciences School of Applied Sciences De Montfort University Leicester LE1 9BH UK

## Abstract

Mast cell amines, platelet-activating factor (PAF), thromboxanes and leukotrienes have been shown to be released during nitric oxide-synthase inhibition in the rat intestine. Mast cells in rat isolated omentum (OMCs) or isolated from the rat peritoneal cavity (PMCs) have been used here to investigate the relationship(s) between these agents. N-nitro-L-arginine methyl ester (L-NAME, 100 μM) caused some degranulation of OMCs, but no enhancement of histamine release from PMCs. PAF (5 μM) and U46619 (1 μM) degranulated OMCs and enhanced histamine release from PMCs. Pre-treatment of the omentum with BN52021 (10 μM) inhibited degranulation of OMCs in response to L-NAME, PAF or U46619. Pretreatment with 1-benzylimidazole (5 or 50 μM) inhibited the effect of L-NAME but not that of PAF. Indomethacin (1 μM) or sodium nitroprusside (10 μM) also inhibited the effects of L-NAME, but nordihydroguaiaretic acid (30 μM) did not. In PMCs BN52021 inhibited PAF-induced, but not U46619-induced, release of histamine. These results suggest that inhibition of nitric oxidesynthase in the omentum by L-NAME allows thromboxanes to release PAF, which in turn degranulates and releases histamine from OMCs.


Mediators of Inflammation 5, 257–261 (1996)

### Inhibition of NO-synthase and degranulation of rat omental mast cells *in vitro*

A. M. Northover[CA] and B. J. Northover

Department of Pharmaceutical Sciences, School of Applied Sciences, De Montfort University, Leicester LE1 9BH, UK

[CA]Corresponding Author

**MAST cell amines, platelet-activating factor (PAF), thromboxanes and leukotrienes have been shown to be released during nitric oxide-synthase inhibition in the rat intestine. Mast cells in rat isolated omentum (OMCs) or isolated from the rat peritoneal cavity (PMCs) have been used here to investigate the relationship(s) between these agents. *N*-nitro-L-arginine methyl ester (L-NAME, 100 μM) caused some degranulation of OMCs, but no enhancement of histamine release from PMCs. PAF (5 μM) and U46619 (1 μM) degranulated OMCs and enhanced histamine release from PMCs. Pre-treatment of the omentum with BN52021 (10 μM) inhibited degranulation of OMCs in response to L-NAME, PAF or U46619. Pre-treatment with 1-benzylimidazole (5 or 50 μM) inhibited the effect of L-NAME but not that of PAF. Indomethacin (1 μM) or sodium nitroprusside (10 μM) also inhibited the effects of L-NAME, but nordihydroguaiaretic acid (30 μM) did not. In PMCs BN52021 inhibited PAF-induced, but not U46619-induced, release of histamine. These results suggest that inhibition of nitric oxide-synthase in the omentum by L-NAME allows thromboxanes to release PAF, which in turn degranulates and releases histamine from OMCs.**

**Key words:** BN52021, Indomethacin, Mast cells, *N*-nitro-L-arginine methyl ester, Nitric oxide-synthase, Nordihydroguaiaretic acid, 1-Benzylimidazole, Platelet activating factor

### Introduction

Degranulation of mast cells occurs during ischaemia/reperfusion injury in both the rat mesentery[1] and small intestine,[2] and it can be enhanced by prior administration of *N*-nitro-L-arginine methyl ester (L-NAME), a NO-synthase inhibitor, or it can be inhibited by prior administration of sodium nitroprusside (SNP), an NO donor.[1] Epithelial permeability to $^{51}Cr$ in rat small intestine is also increased by L-NAME and this is associated with mucosal mast cell degranulation.[3] We have shown recently[4] that the L-NAME-induced increase in microvascular permeability to injected colloidal carbon in the rat small intestine is prevented by pre-treatment with cyproheptadine, a mixed histamine and 5-hydroxytryptamine receptor antagonist. Other workers have shown an increase in albumin leakage in the rat small intestine and colon resulting from co-administration of L-NAME and a lipopolysaccharide. This could be inhibited by prior administration of a cyclooxygenase inhibitor, a thromboxane synthase inhibitor, a platelet activating factor (PAF)-receptor antagonist[5] or a 5-lipoxygenase inhibitor.[6]

In the present work we have investigated the possible relationship(s) between these various inhibitors/antagonists and the co-administration of L-NAME. The study has focused on mast cell degranulation in the presence of such agents. Two methods have been used involving, firstly, mast cells *in situ* in rat omental milky spots (OMCs) and, secondly, mast cells isolated from the rat by peritoneal lavage (PMCs).

### Methods

*Omental mast cells:* Female rats weighing about 250 g were killed by inhalation of chloroform vapour. After opening the abdomen with a midline incision followed by a lateral subcostal incision, the lesser omentum was quickly removed into a beaker of normal saline (NS) and then cut into five pieces of approximately equal size. Each piece was quickly but gently spread out on a glass microscope slide and then placed in a moist atmosphere at 37°C. Specimens were then flooded either with NS or with NS plus a putative antagonist for 5 min. The bathing fluid was then drained away and replaced either by some fresh NS, NS plus antagonist, NS plus

© 1996 Rapid Science Publishers agonist, or NS plus antagonist plus agonist. Incubation continued for a further 15 min. Specimens were then quickly drained, washed briefly with distilled water and covered with a solution of 0.05% toluidine blue in McIlvaine's buffer at pH4 for 8 min. After a further quick wash in distilled water, each specimen was covered with a glass coverslip and viewed on the stage of a microscope at × 100 magnification. This protocol was found during pilot experiments to give good differential staining of mast cells. The numbers of metachromatically (pink/magenta)-stained and darkly blue-stained mast cells associated with each milky spot were counted. The mean number of such mast cells per milky spot in each piece of omentum was then calculated. The numbers of milky spots in each piece of omentum varied from 6 to 51, with most being around 25–35.

Compounds tested as possible agonists were: L-NAME, an NO-synthase inhibitor;[7] PAF, a known inflammatory mediator;[8] U46619, a thromboxane-mimetic;[9] and polymixin B (PMXB) and compound 48/80, which are both known to degranulate mast cells.[10] Compounds used as possible antagonists were: indomethacin, a cyclooxygenase inhibitor;[11] SNP, an NO donor;[12] 1-benzylimidazole (BZI), a thromboxane synthase inhibitor;[13] BN52021, a specific PAF receptor antagonist;[14] and nordihydroguaiaretic acid (NDGA), a 5-lipoxygenase inhibitor.[15]

*Histamine release from peritoneal mast cells:* Female rats were killed as above. Then 40 ml of NS at 37°C was injected into each peritoneal cavity and left for 15 min, massaging the abdomen gently from time to time. Approximately 35 ml of mast cell rich-fluid was recoverable from each animal. After spinning the fluid at 670 × ***g***, the resultant cell pellet was re-suspended in NS and then spun again for a total of three times. Finally the pellet was re-suspended in 5 ml NS, and 0.5 ml aliquots of mast cell-rich suspension were placed in each of 10 tubes. A further 0.4 ml of NS or of NS plus a putative antagonist was then added to each tube. Cell suspensions were incubated at 37°C for 10 min, agitating gently from time to time. Then 0.1 ml of NS or of NS plus putative agonist was added to each tube and incubation continued for a further 15 min, at which time histamine release was stopped by adding 1.5 ml ice-cold NS and placing the tubes in melting ice. After spinning the tubes at 670 × ***g*** for 10 min, two 1 ml aliquots from each supernatant were removed and assayed fluorimetrically for histamine using 1% *o*-phthaldialdehyde (OPT) as a fluorophore.[16]

The residual cell pellet was re-suspended in 2.5 ml NS using a vortex mixer and heated for 10 min to 60°C to release all remaining cellular histamine. After cooling, two 1 ml aliquots of this fluid were assayed for their histamine content as above. Histamine released during incubation at 37°C was expressed as a percentage of the total histamine originally present in the cells, and was corrected for the histamine released under appropriate control conditions. One assay comprises the results obtained from one 0.5 ml aliquot of mast cell-rich suspension. Mast cells from a minimum of three rats were used for each treatment group. The total numbers of assays performed per treatment were between seven and 22. Putative agonists used were PMXB, L-NAME, PAF or U46619. Putative antagonists used were SNP and BN52021.

*Statistics:* Bonferroni's test was used for comparing several groups with one control group.

*Chemicals used:* L-NAME, PMXB, PAF, U46619, BZI, NDGA, compound 48/80, and OPT were all obtained from Sigma Chemical Co. Ltd (Poole, UK); SNP was obtained from David Bull Laboratories (Warwick, UK); indomethacin from Merck, Sharpe & Dohme Ltd (Hoddesdon, UK); toluidine blue (batch 9244890D) from BDH (Poole, UK). BN52021 was a gift from Dr P. Braquet, Institut Henri Beaufour (Le Plessis-Robinson, France). L-NAME, PMXB, compound 48/80, BZI, SNP and indomethacin were used as aqueous solutions, indomethacin being dissolved with the aid of a little $Na_2CO_3$. NDGA and BN52021 were dissolved initially in dimethylsulphoxide, the final concentration of solvent being <0.5%. U46619, as a concentrate in methyl acetate, and PAF, as a concentrate in chloroform, were diluted with a little ethanol before final dilution with NS. OPT was dissolved in analytical grade methanol.

### Results

Treatment of the rat omentum *in vitro* with either PMXB (1 mg/ml) or compound 48/80 (1 μg/ml) (Fig. 1) or L-NAME (100 μM) (Fig. 2) or PAF (5 μM) or U46619 (1 μM) (Fig. 3) significantly reduced the number of metachromatically stained OMCs in the milky spots. Lower concentrations of L-NAME (10 μM) or PAF (0.5 μM) or U46619 (0.1 μM), however, were without a significant effect. OMCs that were either substantially or completely degranulated by such treatments stained darkly blue. The ratio of numbers of metachromatically stained OMCs to darkly blue-stained OMCs (M/DB) in the milky spots was 6.3 ± 0.5 in the control preparations. Significant reductions in this ratio ($p < 0.05$,

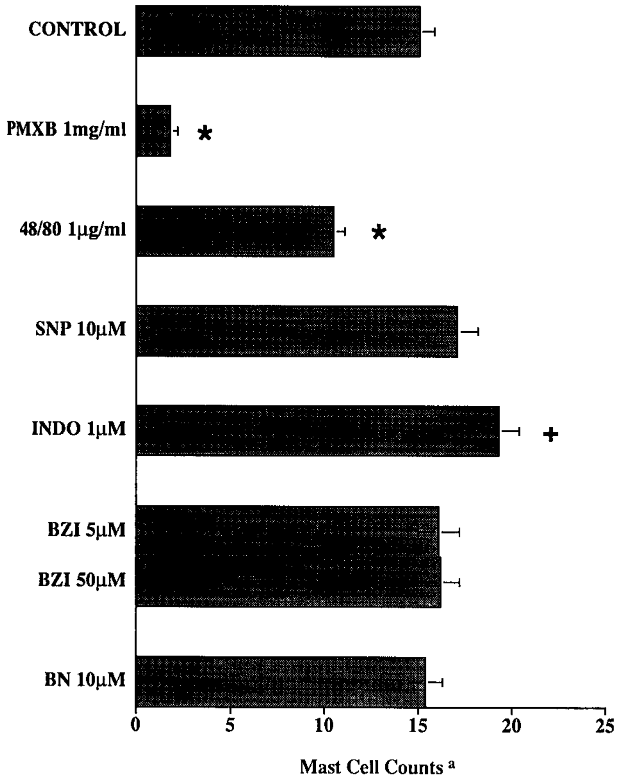

FIG. 1. Effects of various agents on the degranulation of rat omental mast cells. [a]Mast cell counts are the mean numbers ± SEM of metachromatically stained mast cells per omental milky spot. *lower than, +higher than the control value ($p < 0.05$, Bonferroni's test). Abbreviations not used in the text: INDO, indomethacin; BN, BN52021.

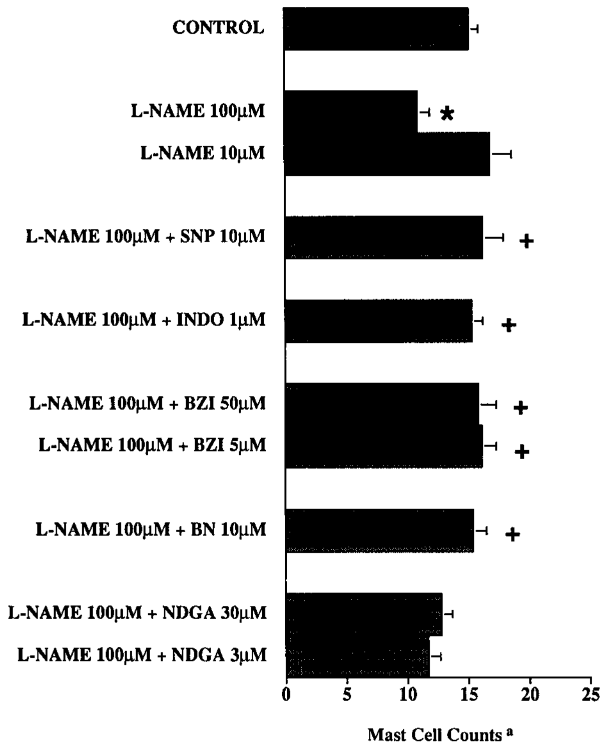

FIG. 2. The degranulating effect of L-NAME on rat omental mast cells, and its modification by various agents. [a]Mast cell counts are the mean numbers ± SEM of metachromatically stained mast cells per omental milky spot. *lower than control value ($p < 0.05$, Bonferroni's test); +higher than the value for L-NAME 100 μM ($p < 0.05$, Bonferroni's test). Abbreviations not used in the text: INDO, indomethacin; BN, BN52021.

Bonferroni's test) occurred after treatment with PMXB (to 0.06 ± 0.02), with 100 μM L-NAME (to 3.4 ± 0.4) and with 5 μM PAF (to 2.8 ± 0.6). Reductions in the M/DB ratio after treatment with compound 48/80 (to 4.0 ± 0.5) or with 1 μM U46619 (to 4.6 ± 0.6) did not quite reach conventional levels of statistical significance. Pre-treating the omentum with SNP (10 μM), with indomethacin (1 μM), with BZI (5 or 50 μM) or with BN52021 (10 μM) significantly reduced the degranulating effects of L-NAME (Fig. 2). Of these antagonists, however, only indomethacin (Fig. 1) gave significant protection against the disappearance of pink/magenta-stained OMCs in NS-treated omenta during incubation at 37°C, and this was accompanied by a significant increase in the M/DB ratio (to 10.5 ± 0.9). There was also a significant increase in the M/DB ratio after pre-treatment with SNP (to 12.6 ± 1.0), but with this drug the increase in the number of metachromatically stained OMCs per milky spot did not reach a conventional level of statistical significance (Fig. 1). Pre-treatment of the omentum with NDGA (3 or 30 μM) did not reduce the effects of L-NAME significantly (Fig. 2). In contrast, pre-treatment with BN52021 (10 μM) significantly reduced the degranulating effects of PAF (Fig. 3), whereas a lower concentration (1 μM) was not significantly effective. Pre-treatment of the omentum with BZI (50 μM) had no effect on the response to PAF. Pre-treatment with BN52021 (10 μM), however, significantly reduced the degranulating effects of U46619 (Fig. 3).

PMCs responded to the addition of PMXB, PAF or U46619 with a significant increase in histamine release (Fig. 4). SNP, on the other hand, significantly decreased the release of histamine from PMCs (Fig. 4). In contrast to its mast cell-degranulating effect in OMCs (see above), L-NAME exerted no significant effect on the release of histamine from PMCs (Fig. 4). However, pre-treatment of PMCs with BN52021 (10 μM) significantly attenuated the histamine-releasing effect of PAF, but not that of U46619 (Fig. 4). In the presence of L-NAME (100 μM) histamine release in response to incubation with PAF or U46619 was increased but only slightly (Fig. 4).

### Discussion

Endogenous NO has been shown to exert

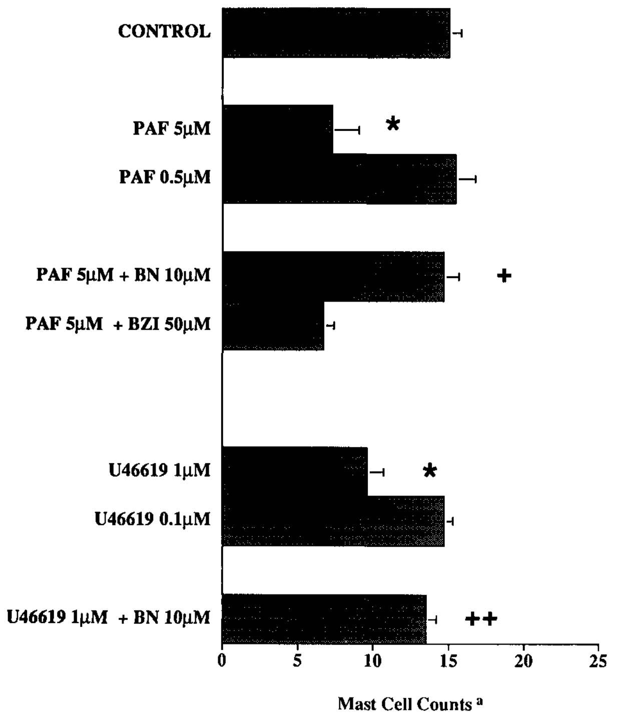

FIG. 3. The degranulating effects of PAF and U46619 on rat omental mast cells, and their modification by BN52021 (BN) or BZI. [a]Mast cell counts are the mean numbers ± SEM of metachromatically stained mast cells per omental milky spot. *lower than control value ($p < 0.05$, Bonferroni's test); +higher than the value for PAF 5 μM ($p < 0.05$, Bonferroni's test); ++higher than the value for U46619 1 μM ($p < 0.05$, Bonferroni's test).

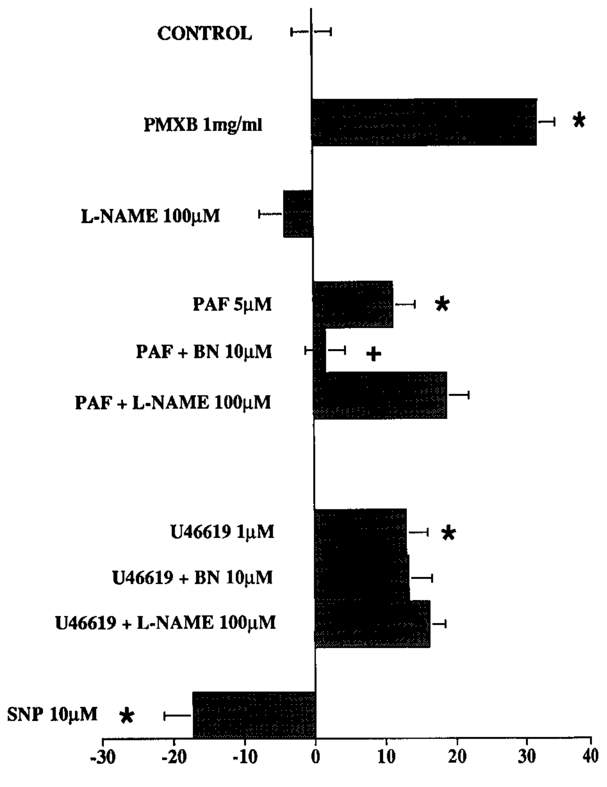

FIG. 4. Effects of various agents on histamine release from rat isolated peritoneal mast cells expressed as percentage increase or decrease ± SEM, after allowance for release in the appropriate controls. *$p < 0.05$, Bonferroni's test, compared with the control value. +$p < 0.05$, Bonferroni's test, compared with the value for PAF 5 μM.

both anti-inflammatory and pro-inflammatory effects, depending upon the circumstances.[17,18] In acute gastrointestinal injury there is substantial evidence to suggest that NO has an anti-inflammatory effect,[17] whereas a pro-inflammatory effect is sometimes more evident during inflammation occurring elsewhere in the body.[18] It has been shown previously that administration of L-NAME, which would be expected to have inhibited NO-synthase, caused leakage of albumin[1] and of injected colloidal carbon[4] from rat small intestines and mesentery that apparently was due to a release of certain mast cell-derived amines. In the present experiments the application of L-NAME caused degranulation of OMCs in the milky spots. In contrast, pre-treatment with SNP, an NO donor, counteracted this degranulation (Fig. 2), suggesting that where OMCs are concerned, both exogenous and endogenous NO can be protective. However, in which of the various omental cell type(s) the synthesis of NO was actually being blocked by added L-NAME remains unknown. In contrast, L-NAME did not increase histamine release from PMCs (Fig. 4). This observation is similar to that of other workers who used $N^G$-monomethyl-L-arginine as an NO-synthase inhibitor.[19] Perhaps resting PMCs have too low a cytoplasmic calcium ion concentration to permit activation of the constitutive form of NO-synthase. If so, then L-NAME would have no enzyme activity to inhibit. However, under certain circumstances, such as during stirring or incubation with lipopolysaccharide, PMCs *in vitro* do respond to treatment with L-NAME or other NO-synthase inhibitors with an enhanced release of histamine.[19] In the present experiments, however, L-NAME only slightly augmented the release of histamine that was produced in PMCs by adding PAF or U46619 (Fig. 4). The reasons for these discrepancies are presently unknown.

Successful antagonism of the degranulating effect of L-NAME on OMCs by pre-incubation with indomethacin, BZI, or BN52021 (Fig. 2) suggests that such degranulation may have been in response to the release of endogenous thromboxane $A_2$ ($TXA_2$) and PAF; but from which of the many cell types to be found within the omentum this release occurs is unknown. It is possible that different cell types may be responsible for secretion of the two agents. Since the degranulating effect of exogenously applied U46619 in OMCs, although not PMCs, was fully blocked by pre-treatment with BN52021, it is likely that the exogenous thromboxane-mimetic was first releasing PAF from the omentum and, therefore, that most of the OMC degranulation seen was due to this PAF, rather than to any direct effect that U46619 also can exert upon OMCs. The degranulating effect of exogenous PAF was blocked by pre-treatment of the omentum with BN52021, as would be expected, but it was not blocked by pre-treatment with BZI. This suggests, therefore, that in the presence of L-NAME there may be a release of $TXA_2$ within the omentum, which in turn releases substantial quantities of endogenous PAF. It may be the degranulating effects of the latter substance, therefore, that were being observed in incubated omenta in the present experiments.

Pre-treatment of the isolated omentum with NDGA in the present work did not affect the extent of L-NAME-induced degranulation of OMCs. This observation contrasts with a previous report that BW A137C, another 5-lipoxygenase inhibitor, could ameliorate the combined effects of L-NAME and lipopolysaccharide in provoking the vascular leakage of albumin,[6] as also did indomethacin, BZI and BN52021.[5] By being able to secrete leukotrienes as well as prostaglandin $D_2$, mucosal mast cells would appear to be phenotypically rather different from the connective tissue variety, since the latter can secrete only minute amounts of leukotrienes.[20] Moreover, OMCs appear to be of the connective tissue type, since Fig. 1 shows that they were degranulated in response to an application of compound 48/80, as were mesenteric and peritoneal mast cells in other earlier experiments,[21] whereas mucosal mast cells were not.[21] However, we are aware that NDGA, unlike BW A137C, has non-specific antioxidant properties. This might account for its lack of effect in the present experiments.

The protective effect of indomethacin on omental mast cells (Fig. 2) is interesting, since inhibiting cyclooxygenase with this drug would be expected to reduce the availability of both prostaglandins and $TXA_2$. Prostaglandins, however, appear to lack a regulatory effect on mast cells.[22] Consequently, only the effect of inhibiting $TXA_2$ production with indomethacin is likely to be evident in the isolated omentum. The overall effect of the drug, therefore, would be a reduced degranulation, an effect similar to that of BZI (Fig. 2), which was used to inhibit the synthesis of $TXA_2$ selectively. It appears that metabolic processes in the mast cell are finely balanced, but they can be pushed by various pharmacological means either towards cellular protection, for example, by inhibiting $TXA_2$ synthesis, or towards cellular damage, by inhibiting NO synthesis. It seems likely that mucosal and omental mast cells react in pharmacologically similar ways. We hope to investigate this hypothesis further, but meanwhile we may conclude that the effects of L-NAME on mast cell degranulation in the rat intestine, mesentery and omentum are rather similar processes, involving a destabilizing influence of endogenous $TXA_2$ and PAF, possibly acting sequentially.

### References

1. Kurose I, Wolf R, Grisham MB, Granger DN. Modulation of ischemia/reperfusion-induced microvascular dysfunction by nitric oxide. *Circ Res* 1994; **74:** 376–382.
2. Boros M, Takaichi S, Masuda J, Newlands GFJ, Hatanaka K. Response of mucosal mast cells to intestinal ischemia-reperfusion injury in the rat. *Shock* 1995; **3:** 125–131.
3. Kanwar S, Wallace JL, Befus D, Kubes P. Nitric oxide synthesis inhibition increases epithelial permeability via mast cells. *Am J Physiol* 1994; **266:** G222–G229.
4. Northover AM, Northover BJ. Rat intestinal mast cell amines are released during nitric oxide synthase inhibition *in vitro*. *Mediators Inflamm* 1996; **5:** 32–36.
5. László F, Whittle BJR, Moncada S. Interactions of constitutive nitric oxide with PAF and thromboxane on rat intestinal vascular integrity in acute endotoxaemia. *Br J Pharmacol* 1994; **113:** 1131–1136.
6. László F, Whittle BJR. Colonic microvascular integrity in acute endotoxaemia: interactions between constitutive nitric oxide and 5-lipoxygenase products. *Eur J Pharmacol* 1995; **277:** R1–R3.
7. Rees DD, Palmer RMJ, Schulz R, Hodson HF, Moncada S. Characterization of three inhibitors of endothelial nitric oxide synthase *in vitro* and *in vivo*. *Br J Pharmacol* 1990; **101:** 746–752.
8. Braquet P, Touqui L, Shen TY, Vargaftig BB. Perspectives in platelet-activating factor research. *Pharmacol Rev* 1987; **39:** 97–145.
9. Lefer AM, Darius H. A pharmacological approach to thromboxane receptor antagonism. *Fed Proc* 1987; **46:** 144–148.
10. Ellis III HV, Johnson AR, Moran NC. Selective release of histamine from rat mast cells by several drugs. *J Pharmacol Exp Ther* 1970; **175:** 627–631.
11. Vane JR. Inhibition of prostaglandin synthesis as a mechanism of action of aspirin-like drugs. *Nature (Lond)* 1971; **231:** 232–239.
12. Gruetter CA, Barry BK, McNamara DB, Gruetter DY, Kadowitz PJ, Ignarro LJ. Relaxation of bovine coronary artery and activation of coronary arterial guanylate cyclase by nitric oxide, nitroprusside and a carcinogenic nitrosoamine. *J Cyclic Nucleotide Res* 1979; **5:** 211–224.
13. Tai H-H, Yuan B. On the inhibitory potency of imidazole and its derivatives on thromboxane synthetase. *Biochem Biophys Res Commun* 1978; **80:** 236–242.
14. Braquet P, Godfroid JJ. PAF-acether specific binding sites: 2. Design of specific antagonists. *Trends Pharmacol Sci* 1986; **7:** 397–403.
15. Tateson JE, Randall RW, Reynolds CH, Jackson WP, Bhattacherjee P, Salmon JA, Garland LG. Selective inhibition of arachidonate 5-lipoxygenase by novel acetohydroxamic acids: biochemical assessment *in vitro* and *ex vivo*. *Br J Pharmacol* 1988; **94:** 528–539.
16. Loeffler LJ, Lovenberg W, Sjoerdsma A. Effects of dibutyryl-3′, 5′-cyclic adenosine monophosphate, phosphodiesterase inhibitors and prostaglandin $E_1$ on compound 48/80-induced histamine release from rat peritoneal mast cells *in vitro*. *Biochem Pharmacol* 1971; **20:** 2287–2297.
17. Kubes P, Wallace JL. Nitric oxide as a mediator of gastrointestinal mucosal injury?—Say it ain't so. *Mediators Inflamm* 1995; **4:** 397–405.
18. Miller MJS, Grisham MB. Nitric oxide as a mediator of inflammation?—You had better believe it. *Mediators Inflamm* 1995; **4:** 387–396.
19. Salvemini D, Masini E, Pistelli A, Mannaioni PF, Vane J. Nitric oxide: a regulatory mediator of mast cell reactivity. *J Cardiovasc Pharmacol* 1991; **17 (Suppl 3):** S258–S264.
20. Foreman JC. Mast cells and basophil leucocytes. In: Dale MM, Foreman JC, Fan T-PD, eds. *Textbook of Immunopharmacology*. London: Blackwell Scientific Publications, 1994; 21–34.
21. Enerbäck L. Mast cells in rat gastrointestinal mucosa. 3. Reactivity towards compound 48/80. *Acta Path et Microbiol Scand* 1966; **66:** 313–322.
22. Salmon JA, Higgs GA. The eicosanoids: generation and actions. In: Dale MM, Foreman JC, Fan T-PD, eds. *Textbook of Immunopharmacology*. London: Blackwell Scientific Publications, 1994; 131–142.

**Received 19 April 1996; accepted in revised form 28 May 1996**
